# Cutaneous metastasis from pleural mesothelioma at site of radiation therapy: Case report and review of the literature

**DOI:** 10.1016/j.jdcr.2024.08.033

**Published:** 2024-09-18

**Authors:** William Abou Shahla, Dana Maria Khoury, Dana Saade

**Affiliations:** Department of Dermatology, American University of Beirut Medical Center, Beirut, Lebanon

**Keywords:** cutaneous metastasis, pleural mesothelioma, radiation therapy, skin disease

## Introduction

Mesothelial tumors, derived from the mesothelium or lining of different internal organs such as the lungs and heart, can be either benign and preinvasive neoplasms or malignant mesotheliomas (MMs). The former group includes well-differentiated papillary mesothelial tumors, adenomatoid tumors, and mesothelioma in situ.^1^ MMs can be localized or diffuse and are divided by the World Health Organization classification system into 3 main histologic types: epithelioid, biphasic, and sarcomatoid. These subtypes play a crucial role in determining the prognosis and treatment strategies for affected patients.^1^ The most common type, pleural mesothelioma, is a rare, aggressive tumor with poor prognosis, often linked to asbestos exposure. Cutaneous metastasis of a malignant mesothelial tumor is relatively uncommon. Three different routes of metastasis have been reported: regional spread through lymphatics, direct extension along surgical scars such as needle tracks, and distant metastasis via hematogenous spread.^2^ This report describes a 68-year-old man who developed cutaneous metastasis following radiation therapy for pleural mesothelioma.

## Case report

**Fig 1:**
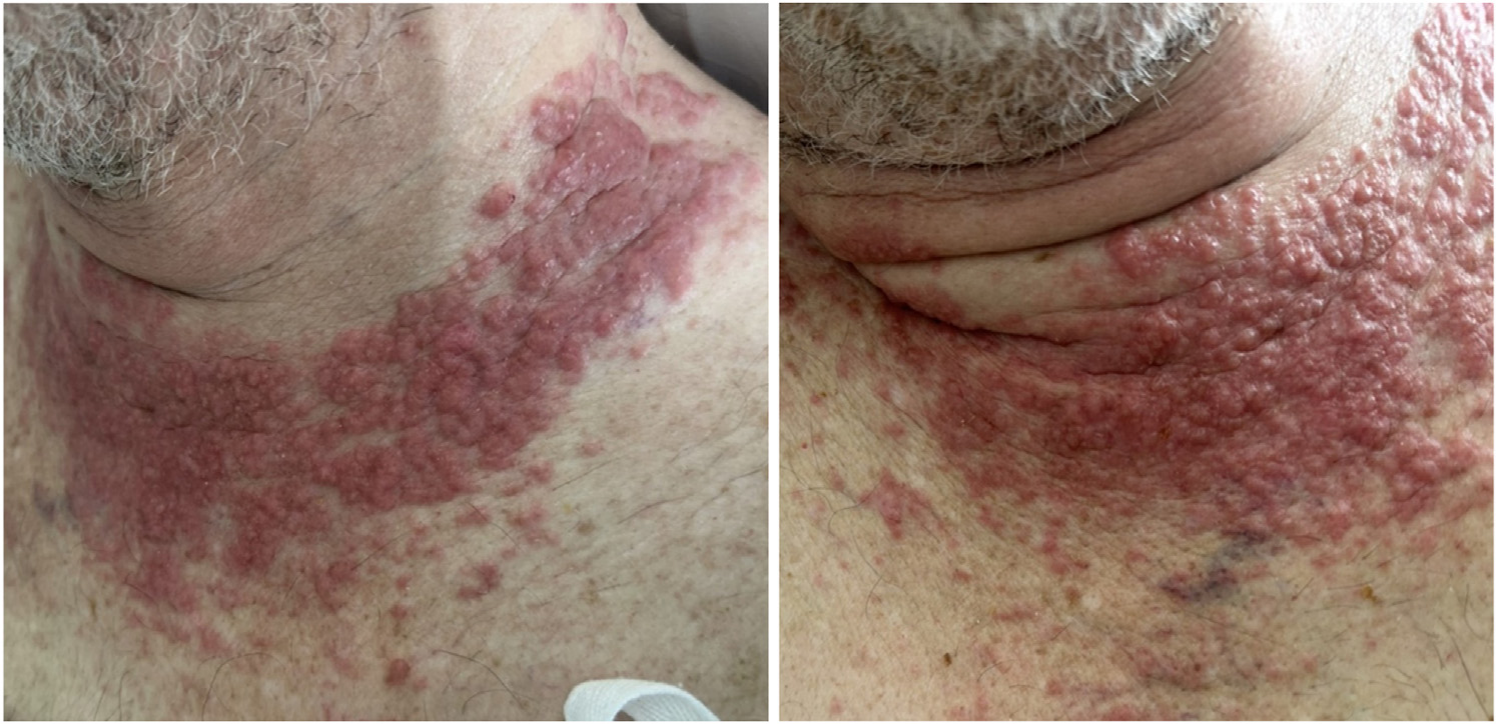
Infiltrated erythematous papules and plaques over the chest and neck.

This is the case of a 68-year-old man, known to have diabetes mellitus, admitted to the intensive care unit with dyspnea and desaturation. He was found to have a subsegmental pleural embolism with superimposed pneumonia. The patient was diagnosed a year ago with right-sided malignant pleural mesothelioma for which he had received chemotherapy and immunotherapy, discontinued 3 months prior to presentation. Palliative radiotherapy (30 Gy in 10 fractions) was then performed to the mediastinum reaching superior vena cava lymph nodes due to progression of disease with a resulting superior vena cava syndrome. During his admission, the patient had an asymptomatic rash over the chest and neck of 2 months duration. It started with a few discrete lesions on the right side of his chest after receiving radiation therapy to the area. The lesions then progressively increased in number to involve the entire surface that was subjected to radiation and some of the surrounding areas. On examination, we could appreciate numerous erythematous, hard, infiltrated papules, some coalescing to form large plaques over the upper part of the chest extending to the lower neck in a necklace pattern (Fig 1). Differential diagnosis included radiation-induced dermatitis, granulomatous dermatoses, and cutaneous metastasis. A punch biopsy was obtained from one of the representative lesions.

**Fig 2:**
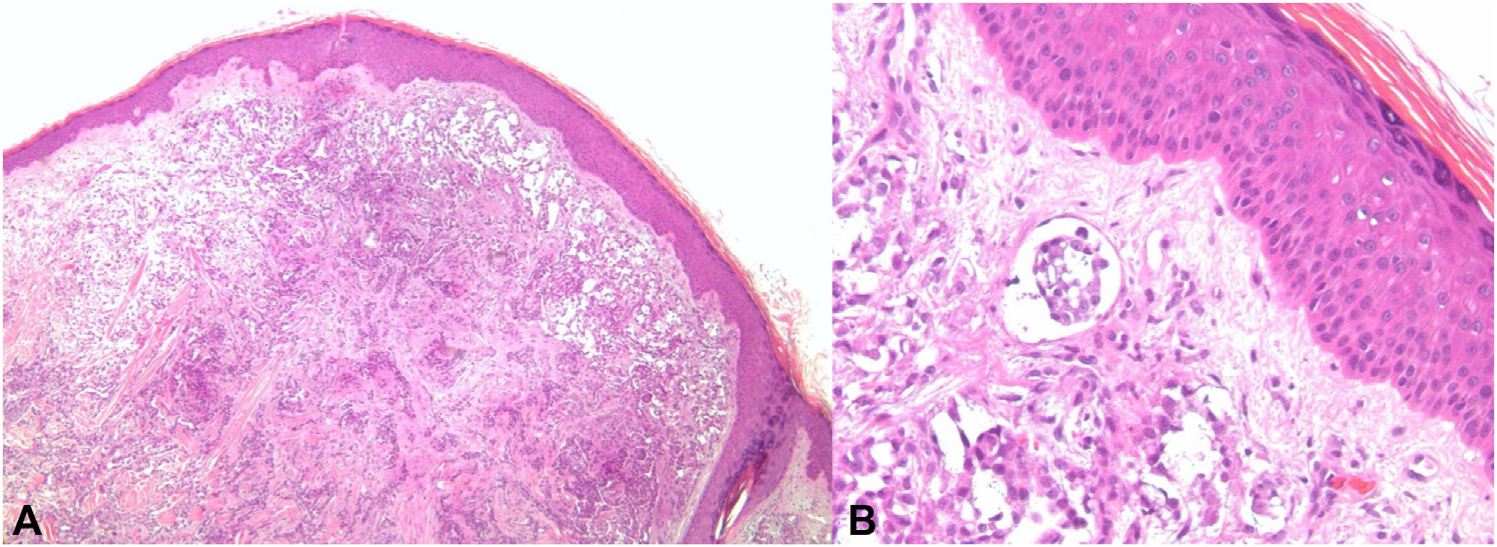
**A,** Nodular proliferation of neoplastic monomorphic cells in the dermis. **B,** Lymphatic invasion.

**Fig 3:**
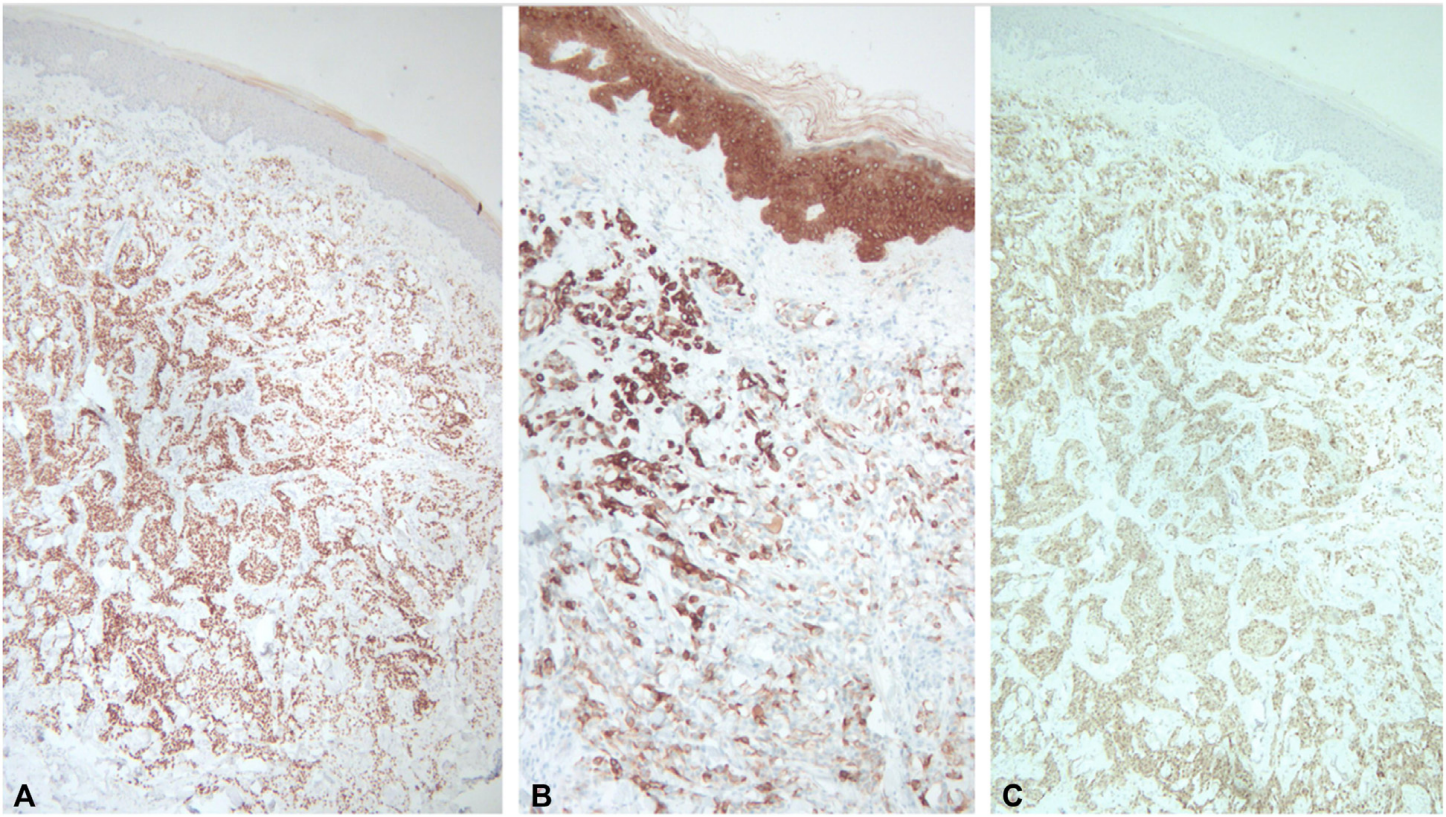
Positive immunohistochemical stain for: **A,** WT1, (**B**) CK 5/6, (**C**) Calretinin. *CK 5/6*, Cytokeratin 5/6; *WT1*, Wilms tumor 1.

Histopathological examination revealed a dermis occupied by a nodular proliferation of neoplastic monomorphic cells with vacuolated cytoplasm and cytologic atypia. At higher magnification, lymphatic invasion was noted (Fig 2). Immunohistochemical studies revealed malignant cells positive for the mesothelial markers cytokeratin 5/6, calretinin, and Wilms tumor 1 (Fig 3). A diagnosis of cutaneous metastasis from known malignant pleural mesothelioma was made. The patient unfortunately passed away shortly afterward.

## Discussion

MM is an aggressive, locally invasive tumor that can metastasize to distant organs, including the skin, though rarely. Cutaneous involvement occurs through 3 distinct routes: regional spread via the lymphatic system, direct extension into surgical scars, and distant metastasis through the bloodstream.^3^ Pleural MM accounts for 85% of reported cases of cutaneous metastasis, while peritoneal MM accounts for 15%. About 60% of pleural MM cases are epithelioid, 23% sarcomatoid, and 17% biphasic.^3^ The most common sites for cutaneous involvement include the face, scalp, and chest, usually present a few months after MM diagnosis, though delayed appearance up to 4 years has been reported.^4^ In our patient, the skin lesions first appeared 8 months after the diagnosis of malignant pleural mesothelioma. Interestingly, mostly confined to the radiation field and its surroundings. This predilection of metastasis to radiation sites has been rarely described in the literature and was named by Shurman et al the “isoradiotropic response.” It is hypothesized that irradiated skin serves as a vulnerable milieu with a dysregulated immune function and impaired lymphatic drainage, allowing malignant cells to thrive. Radiation port cutaneous metastasis has been most frequently reported in primary breast cancer, but also in patients with anogenital, nasopharynx, and gastric tumors treated with radiotherapy.^5^

Diagnosing epithelioid mesothelioma can be challenging, particularly through skin biopsies, due to varied histologic presentations. As a result, colorectal adenocarcinoma, renal cell carcinoma, prostatic adenocarcinoma, angiosarcoma, and lung adenocarcinoma must be considered in the histologic differential diagnosis.^6^ Differentiating adenocarcinoma from epithelioid mesothelioma is challenging, but immunohistochemical studies are helpful in distinguishing these entities. Calretinin, Wilms tumor 1, D2-40, cytokeratin 5/6, thrombomodulin, and human bone marrow endothelium marker-1 are key markers for mesothelioma, while carcinoembryonic antigen, antihuman epithelial antigen, TAG-72, Ep-CAM/Epithelial Specific Antigen, and thyroid transcription factor 1 are markers for lung adenocarcinoma.^7^ A panel of these antibodies can aid in accurate diagnosis. According to the International Mesothelioma Interest Group, a preliminary diagnostic immunohistochemical panel should include 2 mesothelial markers and 2 carcinoma markers to optimize sensitivity and specificity.^8^

Few cases describe distant cutaneous metastasis from primary MM. For instance, Collins et al portrayed 2 cases of MM metastatic to the head and neck region. The first case involved a 64-year-old man with a history of abdominal and thoracic MM developed a rapidly growing lesion on the upper lip, and a 77-year-old woman with malignant pleural mesothelioma presented with a subcutaneous nodule on the forehead. Histopathology showed dermal proliferation of epithelioid cells with cytologic atypia and several mitotic figures. Immunohistochemical stains were positive for mesothelial markers and negative for melanocytic and keratinocytic markers, confirming metastatic mesothelioma.^9^

The management of MMs depends on tumor staging, which determines the feasibility of surgical resection. Systemic therapies are crucial for most cases. Historically, chemotherapeutic agents were reported to have limited results, with many drugs showing response rates under 20% and median survival below 1 year.^10^ Recently, optimism has grown as several cytotoxic agents have shown reproducible responses, improved quality of life, and prolonged survival. They may be used as neoadjuvant or adjuvant therapies. These include platinum-based agents, pemetrexed, raltitrexed, vinorelbine, and vinflunine.^10^ Radiation therapy is mainly used as palliative treatment in metastatic disease.

## Conclusion

As the incidence of MMs rises due to increased asbestos use in developing countries, and improved survival with new treatments, the frequency of reported cutaneous metastases is expected to grow.^11^ The use of radiotherapy in the management of these patients might lead to an “isoradiotropic response” where cutaneous metastasis preferentially appears on irradiated skin. Clinicians should watch for cutaneous involvement in patients with MM, especially on irradiated areas.

## Conflicts of interest

None disclosed.

## References

[1] Dacic S. (2022). Pleural mesothelioma classification-update and challenges. Mod Pathol.

[2] Mori T., Yamamoto T. (2021). Skin metastasis of malignant mesothelioma. An Bras Dermatol.

[3] Ward R.E., Ali S.A., Kuhar M. (2017). Epithelioid malignant mesothelioma metastatic to the skin: a case report and review of the literature. J Cutan Pathol.

[4] Elbahaie A.M., Kamel D.E., Lawrence J., Davidson N.G. (2009). Late cutaneous metastases to the face from malignant pleural mesothelioma: a case report and review of the literature. World J Surg Oncol.

[5] Hoyt B.S., Cohen P.R. (2014). Radiation port cutaneous metastases: reports of two patients whose recurrent visceral cancers presented as skin lesions at the site of previous radiation and literature review. Indian J Dermatol.

[6] Abban C., Viglione M. (2009). Peritoneal mesothelioma presenting as a skin nodule. J Cutan Pathol.

[7] Terada T. (2011). Skin metastasis of pleural epithelioid malignant mesothelioma. Appl Immunohistochem Mol Morphol.

[8] Sinn K., Mosleh B., Hoda M.A. (2021). Malignant pleural mesothelioma: recent developments. Curr Opin Oncol.

[9] Collins K., Nagarajan P., Aung P.P. (2021). Distant cutaneous metastasis of malignant epithelioid mesothelioma. J Cutan Pathol.

[10] Kindler H.L. (2008). Systemic treatments for mesothelioma: standard and novel. Curr Treat Options Oncol.

[11] Carbone M., Yang H., Pass H.I., Taioli E. (2023). Did the ban on asbestos reduce the incidence of mesothelioma?. J Thorac Oncol.

